# Updating vital status by tracking in the community among patients with epidemic Kaposi sarcoma who are lost to follow-up in sub-Saharan Africa

**DOI:** 10.1186/s12885-017-3549-1

**Published:** 2017-09-02

**Authors:** Aggrey Semeere, Esther Freeman, Megan Wenger, David Glidden, Mwebesa Bwana, Micheal Kanyesigye, Fredrick Chite Asirwa, Elyne Rotich, Naftali Busakhala, Emmanuel Oga, Elima Jedy-Agba, Vivian Kwaghe, Kenneth Iregbu, Clement Adebamowo, Antoine Jaquet, Francois Dabis, Sam Phiri, Julia Bohlius, Matthias Egger, Constantin T. Yiannoutsos, Kara Wools-Kaloustian, Jeffrey Martin

**Affiliations:** 1 Infectious Diseases Institute, Makerere University College of Health Sciences, Kampala, Uganda; 2 University of California San Francisco, San Francisco, CA United States; 3 Massachusetts General Hospital, Boston, MA USA; 4 Mbarara University of Science and Technology, Mbarara, Uganda; 5 Indiana University, Indianapolis, IN USA; 6 AMPATH, Moi University, Eldoret, Kenya; 7 Institute of Human Virology, Abuja, Nigeria; 8 London School of Hygiene and Tropical Medicine, University of London, London, UK; 9 University of Abuja Teaching Hospital, Gwagwalada, Nigeria; 10 National Hospital of Abuja, Abuja, Nigeria; 11 INSERM U1219 & ISPED, Université Bordeaux, Bordeaux, France; 12 Lighthouse Trust Clinic, Lilongwe, Malawi; 13 University of Bern, Bern, Switzerland

**Keywords:** Loss to follow-up, Tracking, Tracing, Updating vital status, Survival, Mortality, Kaposi sarcoma, HIV/AIDS, Cancer, Resource-limited settings, Sub-Saharan Africa

## Abstract

**Background:**

Throughout most of sub-Saharan Africa (and, indeed, most resource-limited areas), lack of death registries prohibits linkage of cancer diagnoses and precludes the most expeditious approach to determining cancer survival. Instead, estimation of cancer survival often uses clinical records, which have some mortality data but are replete with patients who are lost to follow-up (LTFU), some of which may be caused by undocumented death. The end result is that accurate estimation of cancer survival is rarely performed. A prominent example of a common cancer in Africa for which survival data are needed but for which frequent LTFU has precluded accurate estimation is Kaposi sarcoma (KS).

**Methods:**

Using electronic records, we identified all newly diagnosed KS among HIV-infected adults at 33 primary care clinics in Kenya, Uganda, Nigeria, and Malawi from 2009 to 2012. We determined those patients who were apparently LTFU, defined as absent from clinic for ≥90 days at database closure and unknown to be dead or transferred. Using standardized protocols which included manual chart review, telephone calls, and physical tracking in the community, we attempted to update vital status amongst patients who were LTFU.

**Results:**

We identified 1222 patients with KS, of whom 440 were LTFU according to electronic records. Manual chart review revealed that 18 (4.1%) were classified as LFTU due to clerical error, leaving 422 as truly LTFU. Of these 422, we updated vital status in 78%; manual chart review was responsible for updating in 5.7%, telephone calls in 26%, and physical tracking in 46%. Among 378 patients who consented at clinic enrollment to be tracked if they became LTFU and who had sufficient geographic contact/locator information, we updated vital status in 88%. Duration of LTFU was not associated with success of tracking, but tracking success was better in Kenya than the other sites.

**Conclusion:**

It is feasible to update vital status in a large fraction of patients with HIV-associated KS in sub-Saharan Africa who have become LTFU from clinical care. This finding likely applies to other cancers as well. Updating vital status amongst lost patients paves the way towards accurate determination of cancer survival.

## Background

Knowledge of survival after a cancer diagnosis is one of the fundamental metrics in cancer epidemiology. Accurate survival estimation requires a representative sample (if not census) of patients diagnosed with a particular malignancy as well as knowledge of vital status in all patients following diagnosis. In resource-rich settings, accurate estimation of cancer survival is achieved by combining data from well-curated cancer registries (to identify the cases) and death registries (to ascertain vital status) [1]. In resource-poor settings, however, estimation of cancer survival is elusive. For example, in sub-Saharan Africa, only four cancer registries are deemed to be high quality by the International Agency for Research on Cancer (IARC) [2] and death registries are limited to one country [3]. Without registries, most attempts at cancer survival estimation in sub-Saharan Africa come from facility-based samples (i.e., clinical care), which not only suffer from uncertain representativeness but also have high rates of patients ceasing to return to care without knowledge of their vital status (a phenomenon termed “lost to follow-up” — LTFU). For example, a recent Ethiopian study of survival of cervical cancer had almost 35% LTFU at 2 years [4]. Because it is unwise to assume that patients with cancer who cease to return to care experience similar survival as those whose vital status is documented, accurate survival estimation in the face of sizeable LTFU is precluded.

One cancer in sub-Saharan Africa that needs a better understanding of its survival is Kaposi sarcoma (KS). Even before the HIV epidemic, KS was among the more common cancers in Africa [5, 6], and it exploded in incidence after HIV appeared [7–9]. During the early HIV epidemic, when there was no therapy, KS was associated with very poor survival [8, 10]; this was apparent even with high LTFU. The advent of antiretroviral therapy (ART) has substantially improved KS survival in resource-replete settings [11–13], and ART is now fortunately more widely available in resource-poor regions [14]. Improved survival in the ART era in resource-rich settings, however, cannot blithely be extrapolated to resource-poor settings. Rather, we must study KS survival directly in Africa if we hope to understand the impact of ART in this region. Unfortunately, accurate estimation of contemporary KS survival in Africa has typically been stymied by high LTFU [15–18].

In an attempt to overcome the problem that LTFU presents for cancer survival estimation in Africa, we developed a process whereby we sought after patients who had become LTFU in order to update their vital status. Tracking lost cancer patients has been rarely performed in sub-Saharan Africa [19–21] and little has been described regarding its success. We sought after a large group of lost patients with HIV-related KS in whom our objective was to determine the overall success in updating vital status, assess the relative contribution of different aspects of the search process; and evaluate key determinants of tracking success.

## Methods

### Overall design

At HIV primary care clinics in 4 countries in sub-Saharan Africa, we identified all patients newly diagnosed with KS via a search of electronic databases. Among these patients with KS, we then used the database to determine who appeared to be LTFU. Among those who appeared to be LTFU, we attempted to update their vital status using manual chart review, telephone calls, and physical tracking in the community.

### Study population

Through a search of the electronic databases that hold clinical records at each site, we identified all HIV-infected adults (≥18 years old) newly diagnosed with KS from January 2009 to December 2012 while receiving primary care at one of 33 ambulatory clinics in Kenya, Uganda, Malawi, and Nigeria. The sites included 26 clinics in a network in western Kenya (Academic Model Providing Access to Healthcare (AMPATH) [22]), one clinic in Uganda (Immune Suppression Syndrome (ISS) Clinic in Mbarara), one clinic from Malawi (Lighthouse Trust in Lilongwe) [23], and two clinics in Nigeria (University of Abuja Teaching Hospital (UATH) and National Hospital of Abuja (NHA)). All these sites participate in the International Epidemiologic Databases to Evaluate AIDS (IeDEA) Consortium. Established in 2005 by the U.S. National Institutes of Health, the IeDEA Consortium has as its main objective the harmonization of data collected by geographically disparate, but representative, cohorts of persons infected with HIV [24, 25]. Each of these clinics is prototypical for HIV primary care in its respective region and administers free ART following national guidelines. At all these clinics, it was routine practice to attempt to obtain patients’ telephone contacts and location of their residences at the time of initial enrollment into care. All sites also had routine procedures available to attempt to contact, by telephone or by physical tracking, patients who had failed to return for clinic visits shortly after they became lost, but scarce resources often prohibited these from occurring.

**Fig. 1:**
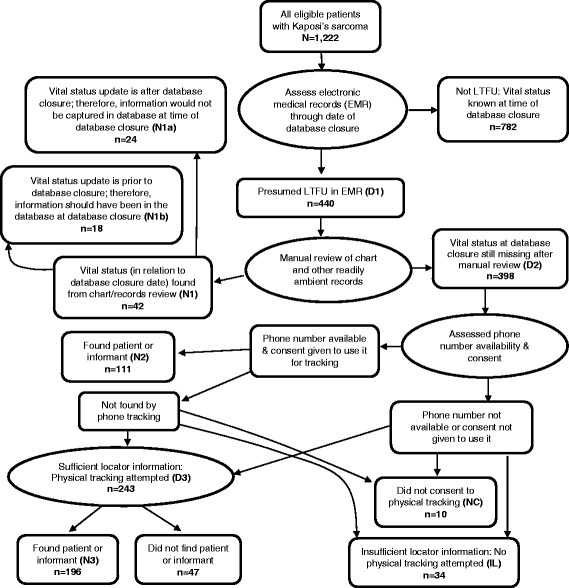
Flow diagram summarizing the logic of the tracking process. Ovals refer to procedures and rectangles refer to outcomes of procedures. Abbreviations (e.g., **N1**) refer to numeric metrics of the process that are referred to in the text. Numbers shown are for the entire population across all four sites. LTFU denotes lost to follow-up

Among the patients with newly diagnosed KS, we then used the respective databases to identify those who were apparently LTFU, as defined by being absent from clinic for at least 90 days at the time of database closure, not known to be dead, and not known to have transferred to another facility (D1 in Fig. 1). For each patient believed to be LTFU, we manually reviewed the paper-based clinic chart for evidence of visits, deaths, or transfers after the last recorded date of being seen that did not get captured in the electronic database. Those who were still LTFU at the time of database closure after this manual inspection were considered truly LTFU (N1a + D2 in Fig. 1).

Approval for this research was granted by each site’s institutional review board.

### Measurements

#### KS

Diagnosis of KS was made during the course of routine clinical care, either by physical examination alone or with histologic confirmation. Both patients who were diagnosed at their first clinic visit and during subsequent care were included.

#### Updating vital status among the LTFU

Among those truly LTFU, we attempted to update vital status, beginning with manual chart review, telephone calls, and finally physical tracking in the community. As noted above, manual chart review had been performed on all apparently lost patients to identify any evidence up to the time of database closure that inadvertently did not get captured in the electronic database. Yet, even if the electronic database had captured all events that had occurred, because there was a delay between database closure and time the search began for a patient believed to be LTFU, manual chart review was also deemed useful because it would detect events (return to care, death, or recorded transfers) that occurred after the time of database closure. Therefore, charts were thoroughly reviewed for their most recent entries that gave any evidence that the patient was either alive or dead. In addition, for those without recent entries, we searched for new phone numbers or geographic locator information that was not present in the electronic database. For telephone tracking, only patients who had provided consent to be called (or to have relatives/friends called) when they enrolled in clinic were contacted. This consent was requested as part of routine clinical care at the sites and not for the purposes of or in anticipation of a research study. Telephone tracking involved using all available phone numbers to call the patient. Calls were attempted at least three times a day on three different days of the week over a period of three weeks until contact was made with either the patient or an informant close to the patient.

Physical tracking was performed for those patients not found by phone, who had consented to be tracked if they had become lost, and who had sufficient geographic locator information (D3 in Fig. 1) to begin a physical search. Again, the documentation of geographic information was done during routine clinical care and did not have any special emphasis in anticipation of research. If there was sufficient locator detail to pursue physical tracking, the tracking was performed by a trained research associate (a “tracker”) for whom the minimal requirements were an intimate understanding of the local dialect, customs, geography and social relationships. Trackers were typically identified amongst existing staff whose current responsibility was, at least in part, to search for lost patients for clinical purposes, namely to bring them back into care. No explicit health care background or education was required; most trackers were some form of community health workers. Prior to commencing the tracking process, the trackers at each site underwent an in-person training conducted by one of the central investigators (A.S. or E.F.) to ensure that standard procedures were followed across sites. Training emphasized maintenance of privacy, avoidance of HIV status disclosure and attentiveness to sensitivity when interacting with HIV-infected patients or their close relatives. For example, trackers used unmarked cars or public transport during the tracking. Importantly, as opposed to the tracking that had previously been done at some of the sites where the goal was to look for as many lost patients as possible and encourage those who were lost to return, the emphasis for the present study was to look for a limited number of lost patients and spend a considerable amount of time, if needed, to find each [26]. Specifically, if the initial attempt was unsuccessful, trackers made at least one additional attempt and, in many instances, made two additional attempts. Tracking was overseen by a local supervisor, but all patients who were difficult to find were discussed with one of the central investigators (either A.S. or E.F.) and consensus was reached prior to deciding to stop tracking a particular patient. Trackers were charged with finding the lost patient, or failing that, a close informant who knew the patient’s vital status. If the tracker found the patient, the date of the encounter was documented. If only an informant who knew the lost patient, was identified, the tracker recorded either the date of death or the most recent date the informant knew the patient was alive. A single informant’s report of death was considered final; there was no attempt to confirm this was another informant, and there were no municipal death registries to cross reference. Searching for multiple patients simultaneously in the same geographic area was encouraged for cost saving.

#### Other measurements

We used the electronic database to obtain age, sex, date of KS diagnosis, date of ART start (if applicable), and date of last clinic visit. We subsequently derived duration since KS diagnosis (time from KS diagnosis to date last seen at clinic) and duration of LTFU (time from last visit to database closure).

### Statistical analysis

For those patients with KS who were LTFU, we first described the success of updating vital status using the three approaches: manual chart review, telephone calls, and physical tracking. Among those patients who were truly LTFU with no recent record of a visit to the clinic upon manual chart review, had sufficient locator information to perform a search, and had provided consent to search for them, we then assessed the independent influence of two factors (duration of being lost and geographic clinic site) on successful tracking. The rationale for examining duration of being lost was to inform programs when, if ever, it becomes too late to search for a lost patient. The rationale for evaluating geographic site was to determine if some aspect of the socio-geographic environment influences success of tracking. The outcome in this analysis was failure to update vital status after telephone and field tracking. The relationships between duration of being lost, geographic site, and failure to locate a lost patient were depicted with risk ratios, which were derived from log binomial regression. In these models, we adjusted for age, sex, duration with KS, and ART status. Multiplicative interaction between duration being lost and geographic site was also evaluated. All analyses were performed using Stata (version 13.1, Stata Corp., College Station, Texas).

## Results

### Identification and characteristics of the population of patients who were LTFU

In total, 1222 patients were diagnosed with KS (32% with biopsy confirmation) during the study period: 678 in Kenya, 173 in Uganda, 314 in Malawi, and 57 in Nigeria. Of these, 440 (D1 in Fig. 1) appeared to be LTFU according to the electronic databases. Manual review of the paper clinic charts, however, revealed that 18 (4.1%) should not have been counted as LTFU (N1b in Fig. 1) but were erroneously missed in the electronic database (Table 1). Therefore, 422 were truly LTFU (N1a + D2 in Fig. 1), and in this population, 65% were men, the median age was 35 years, 73% had started ART, the median CD4+ T cell count was 159 cells/mm^3^, the median duration from KS diagnosis to last visit was 1.4 months, and the median duration between last visit and database closure was 21 months (Table 2).

**Table 1:** Disposition and success of updating vital status through manual record review, phone tracking, and physical tracking amongst patients newly diagnosed with Kaposi sarcoma in four countries in sub-Saharan Africa

	AMPATH - Kenya	ISS - Uganda	Lighthouse- Malawi	UATH & NHA - Nigeria	Overall
**Patients diagnosed with Kaposi sarcoma**	678	173	314	57	1222
**Presumed LTFU in EMR** (D1 in Fig. 1)	249	80	75	36	440
**Reclassification after manual review of LTFU in EMR**					
Not truly LTFU: Vital status determined via manual review (N1b in Fig. 1)	16	1	1	0	18
Truly LTFU but vital status determined by repeat later review of records (N1a in Fig. 1)	11	0	2	11	24
Truly LTFU: Vital status missing after review of all records (D2 in Fig. 1)	222	79	72	25	398
**Disposition of those who appear LTFU in EMR** (D1 in Fig. 1)					
Vital status updated by manual records review	27/249 (11%)	1/80 (1.3%)	3/75 (4.0%)	11/36 (31%)	42/440 (9.6%)
Vital status updated by phone contact alone	83/249 (33%)	10/80 (13%)	3/75 (4.0%)	15/36 (42%)	111/440 (25%)
Vital status updated by physical tracking	124/249 (50%)	47/80 (59%)	22/75 (29%)	3/36 (8.3%)	196/440 (45%)
Vital status not updated: consent available	15/249 (6.0%)	22/80 (28%)	37/75 (49%)	7/36 (19%)	81/440 (18%)
Vital status not updated: consent not available	0/249 (0%)	0/80 (0%)	10/75 (13%)	0/36 (0%)	10/440 (2.3%)
**Disposition of those who were truly LTFU** (N1a + D2 in Fig. 1)					
Vital status updated by manual records review	11/233 (4.7%)	0/79 (0%)	2/74 (2.7%)	11/36 (31%)	24/422 (5.7%)
Vital status updated by phone contact alone	83/233 (36%)	10/79 (13%)	3/74 (4.1%)	15/36 (42%)	111/422 (26%)
Vital status updated by physical tracking	124/233 (53%)	47/79 (59%)	22/74 (30%)	3/36 (8.3%)	196/422 (46%)
Vital status not updated: consent available	15/233 (6.4%)	22/79 (28%)	37/74 (50%)	7/36 (19%)	81/422 (19%)
Vital status not updated: consent not available	0/233 (0%)	0/79 (0%)	10/74 (14%)	0/36 (0%)	10/422 (2.4%)
**Disposition of those truly LTFU not found by manual records review** (D2 in Fig. 1)					
Vital status updated by phone contact alone	83/222 (37%)	10/79 (13%)	3/72 (4.2%)	15/25 (60%)	111/398 (28%)
Vital status updated by physical tracking	124/222 (56%)	47/79 (59%)	22/72 (31%)	3/25 (12%)	196/398 (49%)
Vital status not updated: consent available	15/222 (6.8%)	22/79 (28%)	37/72 (51%)	7/25 (28%)	81/398 (20%)
Vital status not updated: consent not available	0/222 (0%)	0/79 (0%)	10/72 (14%)	0/25 (0%)	10/398 (2.5%)
**Disposition of those truly LTFU who were physically sought after in the community** (D3 in Fig. 1)					
Vital status updated by physical tracking	124/131 (95%)	47/69 (68%)	22/35 (63%)	3/8 (38%)	196/243 (81%)
Vital status not updated	7/131 (5.3%)	22/69 (32%)	13/35 (37%)	5/8 (63%)	47/243 (19%)
**Success of tracking using combination of methods among those who consented and had sufficient information for tracking**					
Records, phone contact & physical tracking^a^	218/225 (97%)	57/79 (72%)	27/40 (68%)	29/34 (85%)	331/378 (88%)
Phone contact & physical tracking^b^	207/214 (97%)	57/79 (72%)	25/38 (66%)	18/23 (78%)	307/354 (87%)

LTFU denotes lost to follow-up; EMR denotes electronic medical records; AMPATH denotes Academic Model Providing Access to Healthcare; ISS denotes Immune Suppression Syndrome; UATH denotes University of Abuja Teaching Hospital and NHA denotes National Hospital of Abuja

^a^ Success of tracking using all information available from the manual records review, telephone, and field tracking amongst those truly LTFU and who gave consent to be sought after. This is (N1a + N2 + N3) / (D1-N1b-NC-IL) in Fig. 1

^b^ Success of tracking using information available from telephone and physical tracking amongst those truly LTFU, not updated by manual review, and who gave consent to be sought after. This is (N2 + N3) / (D2-NC-IL) in Fig. 1

**Table 2:** Characteristics of patients with Kaposi sarcoma who were lost to follow-up in four countries in sub-Saharan Africa (N1a + D2 in Fig. 1)

	AMPATH - Kenya *N* = 233	ISS - Uganda *N* = 79	Lighthouse - Malawi *N* = 74	UATH & NHA - Nigeria *N* = 36	Overall *N* = 422
**Age at last visit, years** ^**a**^	35 (30–42)^b^	32 (29–40)	34 (29–40)	36 (32–42)	35 (29–41)
**Male sex** ^**a**^	62%	68%	78%	51%	65%
**ART in use at last visit**	82%	77%	47%	58%	73%
**CD4+** **T-cells** **/mm** ^**3**^ **at last visit** ^**c**^	126 (39–287)	183 (110–317)	231 (141–387)	259 (177–308)	159 (60–312)
**CD4+** **T-cells** **/mm** ^**3**^ **at last visit** ^**c**^ **, category**					
0–50	28%	17%	6.7%	7.7%	22%
51–100	15%	4.4%	6.7%	7.7%	12%
101–200	23%	30%	27%	23%	24%
201–350	18%	30%	33%	46%	24%
351–500	9.4%	13%	20%	0%	10%
> 500	7.3%	4.4%	6.7%	15%	7.5%
**Duration since KS diagnosis at last visit, months**	0.96 (0–3.5)	1.9 (0.3–4.7)	1.9 (0–8.3)	4.7 (0.6–18.1)	1.4 (0.03–5.1)
**Duration of being lost at database closure, months**	17 (11–22)	30 (19–39)	31 (17–47)	26 (14–46)	21 (13–30)

ART denotes antiretroviral therapy; AMPATH denotes Academic Model Providing Access to Healthcare; ISS denotes Immune Suppression Syndrome; UATH denotes University of Abuja Teaching Hospital; and NHA denotes National Hospital of Abuja

^a^ Age missing for 1 person in AMPATH and 1 person in UATH/NHA; sex is missing for 1 person in UATH/NHA

^b^ median (Interquartile range) unless otherwise noted

^c^ 65% missing CD4 count overall (59% AMPATH, 71% ISS, 80% Lighthouse, and 64% UATH/NHA)

### Feasibility of searching for patients truly LTFU

Among the 422 patients who were truly LTFU (N1a + D2 in Fig. 1), we updated vital status among 331 (78%) (Table 1). Manual chart review was responsible for updating vital status in 24 patients (5.7%), as these patients (N1a in Fig. 1), who had been LTFU as of the date of database closure, re-appeared at clinic shortly after database closure. This occurred at 3 of the 4 participating sites, and at one site (Nigeria) represented a substantial fraction (31%) of the truly LTFU population. Telephone calls were responsible for updating vital status in 111 patients (26%), and physical tracking in the community identified the largest fraction of updated vital status — 196 patients (46%). There were some notable differences between sites in terms of which means of investigation were more useful in updating vital status. For example, in Nigeria, 73% of patients had their vital status updated simply by manual chart review and telephone calls, obviating the need for more expensive physical tracking in the community. In contrast, in Malawi and Uganda, only 7% and 13% of patients, respectively, had their vital status updated by the two inexpensive methods. After eliminating the 24 patients who had re-appeared in clinic after database closure, there remained 398 patients in the truly LTFU population (D2 in Fig. 1), and, of these, 307 (77%) had their vital status eventually updated (Table 1). Similar to the entire truly LTFU population, 111 of the 398 (28%) had their vital status updated by telephone calls, and 196 (49%) were updated by physical tracking in the community.

Because the search for lost patients in this study was done in clinics that were not prospectively selected, there had been no dedicated emphasis on obtaining consent from patients to be sought if they became lost or on optimizing the telephone contacts or geographic detail in the locator information regarding the patient’s community residence. This was apparent in that of the 422 patients who were truly lost, 44 (10%) either did not provide consent (NC in Fig. 1) or did not have sufficient residence locator information for a physical search to be initiated (IL in Fig. 1). Thus, when assessing the entire available LTFU population, we cannot observe just how successful our tracking procedures might have been if we had been working with clinics that had been primed for this activity. To attempt to address this, we next limited the truly LTFU population to the 378 who had provided consent to be sought after and had sufficient geographic locator information for a search to be attempted (i.e., those having potential to be found). In this group, we were able to update vital status in 331 (88%) across all sites, which varied from 68% to 97% between sites (Table 1). In this group of truly LTFU for whom there was some potential of being found, after eliminating those patients whose status was updated through manual chart review, there were 354 patients remaining. Of these 354, we were able to update vital status in 307 (87%) via either telephone contact or physical tracking.

### Determinants of successful tracking by telephone and physical tracking

**Fig. 2:**
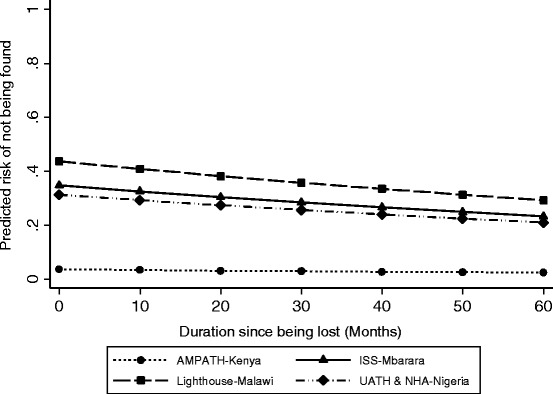
The predicted risk of not being found after phone and physical tracking attempts among patients with Kaposi sarcoma who were lost to follow-up at primary care sites in four countries in sub-Saharan Africa. The prediction is limited to patients who gave consent for tracking and who had sufficient information to attempt physical tracking. Predictions for each site and duration since becoming lost were derived from a generalized linear model and have been adjusted for age, sex, duration since KS diagnosis at last visit, and antiretroviral therapy use. Each prediction represents the mean value of predictions across all patients in the dataset (“marginal” prediction) at their observed values of age, sex, duration since KS diagnosis at last visit, and antiretroviral therapy use. Calculations were performed using the margins command in Stata. AMPATH denotes Academic Model Providing Access to Healthcare in western Kenya; ISS denotes Immune Suppression Syndrome Clinic in Mbarara, Uganda; UATH denotes University of Abuja Teaching Hospital in Abuja, Nigeria; NHA denotes National Hospital Abuja in Abuja, Nigeria

To evaluate the influence of duration of being lost and geographic clinic site on the ability to successfully update the vital status of lost patients by either telephone or physical tracking, we again restricted the population of truly LTFU to the 354 who had provided consent to be searched for, had sufficient geographic locator information for which to base a search, and whose vital status was not updated through manual chart review. In the unadjusted analysis, both duration lost and geographic site were associated with successful tracking (Table 3). After adjustment for age, sex, duration since KS diagnosis, and ART status, duration lost was no longer significant (Table 3 and Fig. 2). There is thus no strong evidence that there is a duration of time that a patient is lost (at least up to the 5 years we evaluated) after which searching should not be attempted. Geographic site, however, remained significant. Compared to AMPATH-Kenya, patients traced at the other three sites were between 8.7 and 12.1 times more likely not to be found (*p* < 0.001). We did not find strong evidence of statistical interaction between geographic location and duration of being lost.

**Table 3:** Determinants of failure to find patients with Kaposi sarcoma who were lost to follow-up in four countries in sub-Saharan Africa. Sample is limited to patients who gave consent for tracking and who had sufficient information to attempt physical tracking (*n* = 354)

	Unadjusted		Adjusted^a^	
	Risk Ratio (95% CI)	*P* value	Risk Ratio (95% CI)	*P* value
**Age at last visit, per 1 year increase**	0.98 (0.95–1.01)	0.21	1.0 (0.97–1.03)	0.87
**Sex**				
Women	Ref.		Ref.	
Men	0.72 (0.42–1.23)	0.23	0. 59 (0.35–1.01)	0.055
**Duration of being lost at database closure, per 1 month increase**	1.03 (1.02–1.05)	<0.001	0.99 (0.98–1.01)	0.48
**Duration since KS diagnosis at last visit, per 1 month increase**	0.96 (0.90–1.02)	0.19	0.94 (0.89–1.00)	0.065
**ART in use at last visit**				
Not on ART	Ref.		Ref.	
On ART	0.40 (0.24–0.68)	0.001	0.68 (0.40–1.14)	0.144
**Site**				
AMPATH-Kenya	Ref.		Ref.	
ISS-Mbarara	8.5 (3.8–19.1)	<0.001	9.7 (4.2–22.2)	<0.001
Lighthouse-Malawi	10.5 (4.5–24.5)	<0.001	12.1 (4.9–29.9)	<0.001
UATH & NHA-Nigeria	6.6 (2.3–19.3)	<0.001	8.7 (2.8–26.6)	<0.001

ART denotes antiretroviral therapy; AMPATH denotes Academic Model Providing Access to Healthcare in western Kenya; ISS denotes Immune Suppression Syndrome Clinic in Mbarara, Uganda; UATH denotes University of Abuja Teaching Hospital in Abuja, Nigeria; and NHA denotes National Hospital Abuja in Abuja, Nigeria

^a^ Adjusted risk ratios were derived using a generalized linear model with a binomial outcome and log link function. All variables are adjusted for all variables in the column

## Discussion

In contrast to resource-rich settings, estimates of cancer survival in sub-Saharan Africa are rare and, even when available, typically have substantial threats to validity. With the ultimate goal of improving the accuracy of the estimation of cancer survival in sub-Saharan Africa, we evaluated the feasibility of searching for a primary care-based sample of patients with HIV-associated KS who had become LTFU from clinical care in 4 different countries. A combination of manual record review, telephone calls, and physical tracking in the community resulted in updating the vital status of a substantial fraction of these lost patients. While duration of being lost did not influence ability to find lost patients, the level of tracking success did vary by geographic site.

Although prior work has recognized the need to search for lost patients in order to accurately estimate cancer survival in sub-Saharan Africa, there is a paucity of data evaluating the feasibility of the tracking process. In the most ambitious prior attempts to estimate cancer survival, performed in Uganda (*N* = 2337 covering 14 different cancers) and Zimbabwe (*N* = 2090 across 15 different cancers), at least 27% (and as many as 49%) of patients in Uganda and 6.6% in Zimbabwe were LTFU [19, 20]. Both studies attempted physical tracking to update vital status but unfortunately did not describe the success of these efforts. Of note, this work was done in the context of cancer registries, which although nominally population-based have had their representativeness critiqued [27, 28]. More recently, Maskew et al., using a primary care-based sample (similar to ours) in South Africa of 247 patients with HIV-related KS who were initiating therapy, observed a rate of LTFU of 13 per 100 person-years. The authors employed “active tracing” of lost patients, but the mechanistic details or success were not reported [29]. Finally, Galukande et al., studying 262 women with breast cancer diagnosed in a tertiary hospital in Uganda, reported that 35% become LTFU [21]. The investigators attempted telephone tracking but again did not describe the outcomes of this effort. Our study builds upon this prior work by searching for what we believe is the largest group of lost patients with a particular cancer in sub-Saharan Africa, deriving these patients from a community-level primary care-based sample, and explicitly delineating the contribution of each of the different elements of the tracking process.

Each component of the search process — manual review of records, telephone calls and physical tracking in the community — did indeed contribute. Because the initial identification of lost patients used electronic records, we hypothesized that clerical errors in recording completed visits in the electronic databases might result in classifying a truly non-lost patient as lost. Furthermore, because some time did pass between the initial identification of apparently lost patients and the time we began to search for them, we recognized that some patients who had been deemed lost at the time of electronic database closure might have subsequently returned to care. Indeed, both of the scenarios occurred, and the manual record review updated an important fraction of nominally lost patients. Telephone calls resulted in updating vital status in about one-quarter of those truly LTFU. Nigeria had the most success with telephone tracking, which is not surprising because the country has one of the highest concentrations of cellphone users in Africa [30]. The third method, physical tracking, found the largest fraction of lost patients. Although we did not set out to evaluate this in the present study, we believe that the mindset we bestowed upon our trackers during our training was critical in their success. That is, we instilled a mentality of looking harder and spending more time on fewer patients — with the goal of determining vital status in each sought patient — than had previously been performed for tracking done during routine clinical care at the respective sites. Most tracking that occurs during routine clinical care is performed in order to bring the lost patient back to care — an individual patient-level perspective. In contrast, we emphasized a group-level perspective: updating vital status in only a small fraction of sought after patients yields little scientifically useful information because we would have no way of being sure whether our found population is representative of all lost patients. Only by updating vital status in a high percentage of the lost can we be certain that we have a representative population. In addition to this training, the attributes that we believe are crucial to trackers’ success include perseverance and intimate knowledge of the local language, culture, and geography.

We found no evidence that duration of being lost was associated with our ability to update vital status. We believe this reflects stable social settings in these regions and the oft-availability of either the patient or his/her informants at the original telephone number or residence. Our finding suggests that searching for lost patients can be performed every 3 to 5 years if resources do not permit more frequently. In contrast, while the overall success of tracking (among those with a potential of being found) was acceptable at each site (≥68%), it was most successful in Kenya. Because site encompasses a number of different constructs, including accuracy of contact information in the records, ease of travel for the trackers, expertise of the trackers, and willingness of the community to provide information to the trackers, we cannot determine which explains the geographic differences. One exception is in Malawi where a substantial percentage of lost patients had already undergone one round of tracking during that clinic’s routine clinical operations such that patients who remained lost at the time we searched for them represented those who were most difficult to update.

The main limitation of our work is that we assessed the feasibility of searching for lost patients in settings which had not been fully prepared for this endeavor. Specifically, information on telephone numbers and residence, as well as consent to search, was obtained during the course of routine care without knowledge that an intense search would later occur for purposes of group-level survival estimation if a patient became lost. Hence, in some instances of lost patients, there was no consent or serviceable locator information, which precluded our ability to search. Therefore, we could not determine how successful the search for the lost can be under optimal conditions in which clinics pay close attention to comprehensive consenting and recording of locator information. We did attempt to estimate success at tracking under optimal conditions by limiting an analysis to lost patients who had given consent to be tracked and had sufficient locator information available. In this group, we were able to update vital status in a very high proportion (88%).

## Conclusions

Our findings have implications for both clinical care venues and cancer epidemiology. Any clinic which routinely records telephone contacts and geographic residence on its patients can follow our described approach and begin searching for its lost patients; outcomes will be dependent upon the completeness and detail of the locator data. Thus, clinics who wish to be highly successful in this regard should begin to comprehensively record and update locator information using standardized protocols and consent all patients to be searched for if they become lost. Such recording can be done inexpensively. In addition to searching for lost patients to update vital status, clinics can also benefit from asking patients who volitionally drop out of care (or their close informants) the reasons why they left. For cancer epidemiology, the ability to update vital status, especially in a primary case-based sample of cancer cases, paves the way towards accurate estimation of cancer survival. While our data was generated for patients with HIV-related KS, we strongly believe our inferences extend to other cancers. We therefore urge cancer epidemiologists to collaborate with sentinel primary care clinics in their regions to identify cohorts of cancer patients, search for the lost patients amongst them, and ultimately make accurate estimates of cancer survival. We believe that such collaborations are much more achievable and will yield actionable estimates of cancer survival than are attempts to emulate the more pristine population-based cancer registries of resource-rich areas.

## Abbreviations

AIDS: Acquired immune deficiency syndrome
AMPATH: Academic model providing access to healthcare
ART: Antiretroviral therapy
HIV: Human immunodeficiency virus
IeDEA: International epidemiologic databases to evaluate AIDS
ISS: Immune suppression syndrome clinic
KS: Kaposi sarcoma
LTFU: Loss to follow-up
